# Previously treated latent tuberculosis infection is associated with less severe acute COVID-19: a cohort study

**DOI:** 10.1136/bmjresp-2024-003003

**Published:** 2025-10-13

**Authors:** Katie Scandrett, Scott Pallett, Yemisi Takwoingi, Adam F Cunningham, Martin Dedicoat, Matthew K O’Shea

**Affiliations:** 1 Department of Applied Health Sciences, College of Medicine and Health, University of Birmingham, Birmingham, UK; 2 NIHR Birmingham Biomedical Research Centre, Birmingham, UK; 3 Centre of Defence Pathology, Royal Centre for Defence Medicine, Birmingham, UK; 4 St George’s University of London Institute for Infection and Immunity, London, UK; 5 Department of Immunology and Immunotherapy, College of Medicine and Health, University of Birmingham, Birmingham, UK; 6 Department of Infection, University Hospitals Birmingham NHS Foundation Trust, Birmingham, UK

**Keywords:** Tuberculosis, Viral infection

## Abstract

**Introduction:**

There is significant potential for respiratory infections, such as tuberculosis (TB) and COVID-19, to overlap but little is known about such co-infection. We aimed to study the impact of active TB and latent TB on the incidence of severe COVID-19 in a large cohort of individuals in a setting of low TB endemicity.

**Methods:**

Clinical data of patients admitted to hospital with acute SARS-CoV-2 were merged with a database of patients with a history of previous or current active TB, latent TB or healthy controls. We assessed the incidence of COVID-19 in these groups, length of hospital stay, admission to the intensive care unit (ICU) and in-hospital mortality.

**Results:**

COVID-19 incidence among individuals with current active TB was 6.2% (12/194) and previous active TB 0.67% (30/4496). In contrast, the incidence in previously treated latent TB was 0.09% (4/4542) and among TB contacts 0.24% (34/13 391). There were similar rates of ICU admission and mortality among individuals with COVID-19 and current active TB, TB contacts and other patients. No individuals with previously treated latent TB and COVID-19 were admitted to the ICU or died.

**Conclusions:**

Individuals with a history of latent TB seem to be at reduced risk of severe COVID-19 and have better outcomes than those with active TB and even uninfected controls. Further studies are required to understand the mechanistic basis of this observation.

**WHAT IS ALREADY KNOWN ON THIS TOPIC**

- Reported outcomes for co-occurrence of latent tuberculosis (TB) and COVID-19 have been limited, with some early evidence suggesting possible immunomodulation between pathogens.

**WHAT THIS STUDY ADDS**

- Patients with previously treated TB infection were less likely to be admitted with COVID-19 to hospital, admitted to ICU or die than those with a history of TB disease, TB contacts or patients with no known history of TB.

**HOW THIS STUDY MIGHT AFFECT RESEARCH, PRACTICE OR POLICY**

- Trained immunity induced by previous TB infection may reduce risk of developing severe COVID-19.
- Further work to assess the relative impact among different socioeconomic and demographic groups and among different COVID-19 strains would provide greater granularity.

## Introduction

*Mycobacterium tuberculosis* (*Mtb*), the causative agent of tuberculosis (TB), is estimated to infect around 25% of the world’s population, cause 10.8 million cases of TB and 1.3 million deaths per year.^1^ Since its emergence in December 2019, SARS-CoV-2 has caused over 777 million confirmed cases and 7.09 million deaths.^2 3^

The COVID-19 pandemic reversed recent improvements in TB control, resulting in an 18% reduction in TB case notifications and 20% fall in patients receiving treatment.^1^ It has been estimated that there were almost 7000 excess deaths from TB in the WHO European Region between 2020–2022 of the COVID-19 pandemic.^4^ The effect of the pandemic on *Mtb* transmission remains controversial. The use of face masks, social distancing and restrictions in physical mobility may have reduced transmission and decreased TB notification.^5^ In contrast, the increases in global and regional TB incidence and mortality seen shortly after notifications declined could have been due to increased transmission and reduced access to care.^6^ However, beyond the impact on TB control programmes, little is understood about the interaction between *Mtb* and SARS-CoV-2, despite their potential for co-infection and crosstalk.^5^

There are conflicting reports on the impact of active TB disease (ATB, defined as a pathological state of *Mtb* infection which results in symptomatic disease) and COVID-19 co-infection on clinical outcomes.^7^,^9^ The interaction between latent TB infection (LTBI, defined as evidence of immune sensitisation to *Mtb* antigens in the absence of features of symptomatic disease) and COVID-19 has been much less studied, but some evidence suggests immunomodulation between pathogens.^10^ The ability of natural mycobacterial infection to induce trained immunity is unknown and given the global incidence of these pathogens, a better understanding of the interplay between *Mtb* and SARS-CoV-2 is important. The aim of this study was to examine the impact of ATB and LTBI on the incidence of severe COVID-19 (defined as individuals requiring hospital admission) in a large cohort of individuals in a setting of low TB endemicity.

## Methods

The clinical data of patients admitted with a new diagnosis of SARS-CoV-2 to a large UK hospital (University Hospitals Birmingham National Health Service Foundation Trust) between 24 February 2020 and 30 October 2020 were extracted from the PIONEER database (excluding existing positive patients and hospital-acquired infections). PIONEER is one of the seven Health Data Research UK’s designated UK-based hubs to facilitate health data use for research. It is a research database and analytical environment that links routinely collected health data across community, ambulance and hospital healthcare providers.^11^ These data were merged with a database of patients with a history of previous treatment for ATB or LTBI, patients currently receiving treatment for ATB or LTBI and TB contacts (individuals negative for ATB or LTBI following screening after contact with an index ATB case; TB contacts are considered to be a matched healthy control group in this study) in Birmingham, collected between 1 January 2011 and 30 October 2020 (Dendrite database, Dendrite Clinical Systems, Reading, UK). The Dendrite database is a web-enabled data capture system which records all new ATB and LTBI cases in Birmingham.^12^

After linkage between these datasets, the data were anonymised and interrogated through a secure Trusted Research Environment. Demographic and clinically important characteristics were extracted. Categorical covariates were described using numbers and percentages and continuous covariates were described using the mean and SD or median and IQR, as appropriate. We calculated the incidence of hospital recorded COVID-19 in ATB, LTBI and TB contacts using data from the Dendrite database as the corresponding denominators over 8 months of the study period. As a comparison, we calculated the overall incidence of hospital recorded COVID-19 using all documented cases during the study period and the estimated population served by the Trust as the denominator. We investigated length of hospital stay, whether patients were admitted to the intensive care unit (ICU), length of stay in ICU and the number of in-hospital deaths. Where appropriate, characteristics of TB contacts, all other non-TB hospital recorded COVID-19 cases and those from the Dendrite database were included for comparison.

### Patient and public involvement

Patients and the public were not involved in the development or analysis, of this study.

## Results

We identified a total of 46 patients with a history or currently receiving treatment for ATB or LTBI who were admitted with COVID-19. Of these, 42 individuals had ATB (30 history and 12 current ATB). Four patients previously received preventive treatment for LTBI.

**Figure 1:**
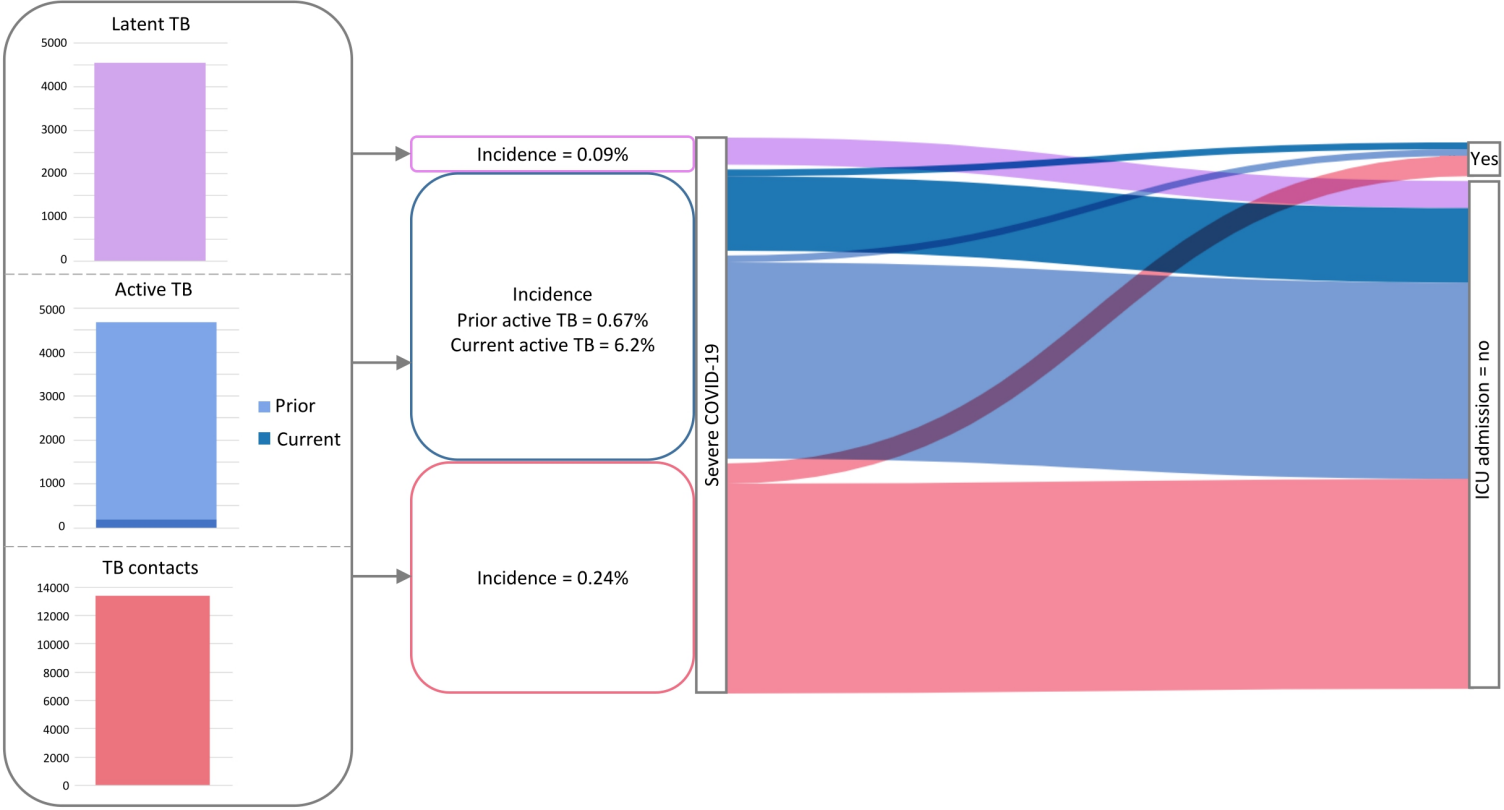
Incidence of severe COVID-19 and admission to ICU among patients with ATB or LTBI compared with TB contacts (February-October 2020). The data demonstrate the incidence of pre-vaccine severe COVID-19 (requiring hospital admission) among patients with prior exposure to TB. Those with severe COVID-19 with a diagnosis of LTBI are represented in purple (n*=*4/4542), those with ATB in blue (n*=*12/194 with currently receiving TB treatment; n=30/4496 with previously treated TB) and TB contacts in red (n=34/13,931). The alluvial plot subsequently visualises incidence of ICU admission among these groups. ATB, active TB; ICU, intensive care unit; LTBI, latent TB infection; TB, tuberculosis.

The Dendrite database contains 4690 known ATB cases, among which 194 were current ATB and 4496 were previously treated cases. The COVID-19 incidence among these groups was 6.2% (12/194) and 0.67% (30/4496), respectively (figure 1). The corresponding incidence for those with previously treated LTBI was 0.09% (4/4,542). We also identified 34 TB contacts with COVID-19. The incidence of hospital recorded COVID-19 among this group was 0.24% (34/13,931). In total, 4102 patients were recorded as having COVID-19, giving an incidence of 0.36% (4102/1 140 525).

The COVID-19 positive ATB individuals were older than the other cohorts. Gender and ethnicity were similar across the groups (table 1A). Previously treated ATB patients had higher co-morbidity counts, with higher rates of chronic kidney disease, diabetes and hypertension.

**Table 1:** Demographic characteristics, clinical characteristics and COVID-19 severity. (A) Demographic features of ATB cases (currently receiving TB treatment (current ATB) and previously treated (previous ATB)) and contacts admitted to hospital with COVID-19. (B) Clinical characteristics of TB and COVID-19 patients and detail of COVID-19 severity.

(A) Demographic characteristics							
Characteristic		COVID-19+ current ATB n=12 patients	COVID-19+previous ATB n=30 patients	All ATB patients n=4690 patients	All LTBI patients n=4542 patients	COVID-19+ TB contacts n=34 patients	All TB contacts n=13 931 patients
Age (years) Median (IQR)		59 (52, 72.5)	69 (60, 80)	47 (36, 64)	35 (26, 45)	53.5 (41, 69)	28 (19, 42)
Male n (%)		9 (75)	16 (53)	2242 (59)	2248 (53)	18 (53)	6112 (44)
Ethnic origin* n (%)	Asian	0 (0)	2 (7)	25 (1)	27 (1)	1 (3)	85 (1)
	Black	1 (8)	1 (3)	1046 (25)	963 (23)	3 (9)	1555 (14)
	Indian subcontinent	3 (25)	14 (47)	1968 (46)	2273 (55)	16 (46)	6606 (57)
	White	2 (17)	2 (7)	662 (16)	594 (14)	4 (11)	2300 (20)
	Other	2 (17)	3 (10)	550 (13)	312 (8)	1 (3)	968 (28)

(B) Key clinical characteristics and COVID-19 severity					
Characteristic		COVID-19+ current ATB n=12 patients n=12 adm†	COVID-19+ previous ATB n=30 patients* n=36 adm†	COVID-19+ TB contacts n=34 patients* n=36 adm†	All other patients‡ n=4102 patients* n=4380 adm†
Obese† n (%)		2 (17)	3 (8)	7 (19)	397 (9)
Pre-existing comorbidities† n (%)	Asthma	1 (8)	9 (25)	5 (14)	501 (11)
	Cancer	0 (0)	0 (0)	2 (6)	438 (10)
	CVA	0 (0)	1 (3)	1 (3)	170 (4)
	CKD	2 (17)	16 (44)	7 (19)	890 (20)
	COPD	4 (33)	3 (8)	3 (8)	644 (15)
	Dementia	0 (0)	3 (8)	2 (6)	671 (15)
	Diabetes	6 (50)	23 (64)	13 (36)	1362 (31)
	Hypertension	4 (33)	24 (67)	9 (25)	2331 (53)
	IHD	2 (17)	20 (56)	8 (2)	1007 (23)
	ILD	0 (0)	2 (6)	0 (0)	176 (4)
Comorbidity count†§ n (%)	0	3 (25)	3 (8)	8 (22)	789 (18)
	1	3 (25)	5 (14)	16 (44)	875 (20)
	2	1 (8)	2 (6)	3 (8)	953 (22)
	3	4 (3)	7 (19)	1 (3)	859 (20)
	4	0 (0)	14 (39)	5 (14)	544 (12)
	5	0 (0)	3 (8)	3 (8)	252 (6)
	6	1 (8)	2 (6)	0 (0)	87 (2)
	7	0 (0)	0 (0)	0 (0)	18 (0.4)
	8	0 (0)	0 (0)	0 (0)	3 (0.1)
ICU adm† n (%)		1 (8)	1 (3)	3 (8)	364 (8)
ICU LOS†¶ median hours (IQR)		–	–	–	244 (103–478.5)
Hospital LOS† Median hours (IQR)		133.5 (96.5–167)	158.5 (100.5–338.5)	118.5 (45.5–219)	183 (56–398)
Died in hospital* n (%)		2 (17)	5 (14)	3 (9)	1063 (24)

(A) COVID-19-negative individuals from the Dendrite database are included for comparison. Demographics of the four LTBI and COVID-19 patients are not included; these patients were aged 41 years, 57 years, 64 years and 77 years and three of the four were female. (B) TB contacts with hospital recorded COVID-19 infection and all other hospital recorded COVID-19 cases were included for comparison. None of the four LTBI and COVID-19 patients were admitted to ICU, and none died while in hospital.

* Numbers represent distinct patients.

† Some patients had multiple hospital admissions. Numbers represent distinct admissions rather than distinct patients because values can change between admissions.

‡ Not including TB cases and TB contacts. Includes LTBI patients.

§ Sum of the pre-existing comorbidities.

¶ Raw hospital LOS (hours) data due to small numbers: current ATB=146; previous ATB=296; TB contacts=40, 61, 738.

Adm, admission; ATB, active TB; CKD, chronic kidney disease; COPD, chronic obstructive pulmonary disease; CVA, cerebrovascular accident; ICU, intensive care unit; IHD, ischaemic heart disease; ILD, interstitial lung disease; LOS, length of stay (hours); LTBI, latent TB infection; TB, tuberculosis.

There were similar rates of admission to the ICU and mortality among individuals with COVID-19 and current ATB, TB contacts and other patients, with a trend to lower rates of ICU admission in the previously treated ATB group and lower mortality in the TB contacts (figure 1B). None of the individuals with previously treated LTBI and COVID-19 were admitted to the ICU or died.

## Discussion

During the period of this study, the rate of confirmed COVID-19 cases in Birmingham was 1567/100 000 population while the average annual rate of new TB notifications (active disease) was 18/100 000.^13 14^ We have identified an association between TB and the incidence of severe COVID-19 in a large cohort of individuals that is dependent on TB infection status. The incidence of COVID-19 hospitalisation among individuals with previously treated LTBI was several-fold less than for those with ATB disease. Furthermore, when compared with individuals who had previously received preventive treatment for LTBI, patients with ATB had more severe COVID-19 (as indicated by ICU admission and mortality). Individuals in the ATB groups were generally older, which may be a confounder as age is an independent risk factor for severe COVID-19.^15^

The rate of COVID-19 hospitalisation among TB negative contacts was similar to that of the wider TB negative population. The inclusion of TB contacts is a valuable control group as these individuals frequently share the same ethnicity, social background and other risk factors with an index ATB case.

Our findings that, when compared with previously treated LTBI, ATB is associated with increased risk of severe COVID-19 and higher morbidity and mortality are similar to most other reports.^7 8^ The proposed reasons for this include reduced polyfunctional capacity of CD4+T lymphocytes and impaired IFN-γ responses to SARS-CoV-2 specific antigens.^5 10 16^

In contrast, individuals with a history of LTBI seem to be at reduced risk of severe COVID-19 and have better outcomes than both those with ATB and even uninfected populations (LTBI-negative healthy controls). A recent study using large epidemiological datasets in 24 European countries found that higher LTBI prevalence was consistently associated with significantly lower COVID-19 incidence and mortality among countries with similar confounders.^17^

We have used the terms ATB and LTBI for different states of TB in this study. It should, however, be noted that in recent years there has been increasing recognition that while the binary active-latent concept of TB has facilitated the development and improvement of programmatic management of TB, it is perhaps an oversimplistic model that fails to support further control measures. As such, a concept of a spectrum of TB, including latent *Mtb* infection, incipient TB, subclinical TB and clinical/active disease, has gained prominence.^18 19^ This is a complex and evolving area and highlights variability in macroscopic pathology, infectiousness and symptoms and signs of TB. Recent attempts to develop a new international consensus framework to better define TB have been reported.^20^

The mechanism for this possible protective effect remains unclear. A recent study in a murine model of pre-existing *Mtb* infection at the time of exposure to SARS-CoV-2 showed a significant reduction in early viral replication in the lung and that this *Mtb*-driven protection increased with higher bacterial doses.^21^ Alternatively, it has been suggested that the protective effect induced by *Mtb* could be due to mycobacterial-induced trained immunity, the feasibility of which has recently been shown with heat-killed *Mtb*.^22^ As such, LTBI may be a more relevant correlate of trained immunity than BCG vaccination status and may induce persistent heterologous protective immunity.^17^ In contrast, trained immunity conferred by BCG is thought to be relatively short-lived and wanes within 5 years in the absence of revaccination or re-exposure.^17^

There are limitations to this study. Due to the datasets available, we were not able to determine the interval between ATB or LTBI treatment and COVID-19 diagnosis, previous community COVID-19, prior BCG vaccination or whether patients were admitted elsewhere. There is the possible confounder of bias in decisions to hospitalise individuals with ATB due to concern over comorbidities, although this is unlikely due to the pressures on healthcare capacity at that time, necessitating clinical need as the main criterion. In addition, due to the low number of COVID-19 hospital admissions among ATB and LTBI patients, it was not possible to perform statistical modelling and therefore our analyses are descriptive. However, we consider that these constraints do not detract from the overall message due to the very apparent differences identified between the groups.

We suggest that further work in this area could include extending both the period of analysis to assess whether there were COVID-19 strain-specific effects in this group and also geography, to assess the possible impact of socioeconomic and demographic factors.

## Data Availability

Data are available upon reasonable request.

## References

[1] (2024). Global tuberculosis report 2024.

[2] Zhu N, Zhang D, Wang W (2020). A Novel Coronavirus from Patients with Pneumonia in China, 2019. N Engl J Med.

[3] WHO coronavirus (COVID-19) dashboard. https://covid19.who.int/table.

[4] (2024). Tuberculosis surveillance and monitoring in Europe 2024 – 2022 data. Copenhagen: WHO regional office for Europe and Stockholm: European centre for disease prevention and control: European centre for disease prevention and control, WHO regional office for Europe.

[5] Dheda K, Perumal T, Moultrie H (2022). The intersecting pandemics of tuberculosis and COVID-19: population-level and patient-level impact, clinical presentation, and corrective interventions. Lancet Respir Med.

[6] Falzon D, Zignol M, Bastard M (2023). The impact of the COVID-19 pandemic on the global tuberculosis epidemic. Front Immunol.

[7] Song W-M, Zhao J-Y, Zhang Q-Y (2021). COVID-19 and Tuberculosis Coinfection: An Overview of Case Reports/Case Series and Meta-Analysis. Front Med (Lausanne).

[8] Aggarwal AN, Agarwal R, Dhooria S (2021). Active pulmonary tuberculosis and coronavirus disease 2019: A systematic review and meta-analysis. PLoS One.

[9] Canetti D, Antonello RM, Saderi L (2022). Impact of SARS-CoV-2 infection on tuberculosis outcome and follow-up in Italy during the first COVID-19 pandemic wave: a nationwide online survey. Infez Med.

[10] Flores-Lovon K, Ortiz-Saavedra B, Cueva-Chicaña LA (2022). Immune responses in COVID-19 and tuberculosis coinfection: A scoping review. Front Immunol.

[11] Gallier S, Price G, Pandya H (2021). Infrastructure and operating processes of PIONEER, the HDR-UK Data Hub in Acute Care and the workings of the Data Trust Committee: a protocol paper. *BMJ Health Care Inform*.

[12] Andrews F, Welch S, Scandrett K (2023). Outcomes of TB contact tracing and predictors of success: a 10-year retrospective cohort analysis in Birmingham, UK. Int J Tuberc Lung Dis.

[13] (2020). Health of the region 2020: addressing health and wellbeing inequalities and the impacts of COVID-19 in the West Midlands.

[14] (2021). TB incidence and epidemiology in England, 2021.

[15] Romero Starke K, Reissig D, Petereit-Haack G (2021). The isolated effect of age on the risk of COVID-19 severe outcomes: a systematic review with meta-analysis. BMJ Glob Health.

[16] Riou C, du Bruyn E, Stek C (2021). Relationship of SARS-CoV-2-specific CD4 response to COVID-19 severity and impact of HIV-1 and tuberculosis coinfection. J Clin Invest.

[17] Singh S, Kishore D, Singh RK (2022). Higher BCG-induced trained immunity prevalence predicts protection from COVID-19: Implications for ongoing BCG trials. *Clin Transl Discov*.

[18] Barry CE, Boshoff HI, Dartois V (2009). The spectrum of latent tuberculosis: rethinking the biology and intervention strategies. Nat Rev Microbiol.

[19] Dheda K, Barry CE, Maartens G (2016). Tuberculosis. Lancet.

[20] Coussens AK, Zaidi SMA, Allwood BW (2024). Classification of early tuberculosis states to guide research for improved care and prevention: an international Delphi consensus exercise. Lancet Respir Med.

[21] Baker PJ, Amaral EP, Castro E (2023). Co-infection of mice with SARS-CoV-2 and *Mycobacterium tuberculosis* limits early viral replication but does not affect mycobacterial loads. Front Immunol.

[22] Minute L, Bergón-Gutiérrez M, Mata-Martínez P (2024). Heat-killed Mycobacterium tuberculosis induces trained immunity in vitro and in vivo administered systemically or intranasally. *iScience*.

